# Noise Robustness Low-Rank Learning Algorithm for Electroencephalogram Signal Classification

**DOI:** 10.3389/fnins.2021.797378

**Published:** 2021-11-24

**Authors:** Ming Gao, Runmin Liu, Jie Mao

**Affiliations:** ^1^College of Sports Science and Technology, Wuhan Sports University, Wuhan, China; ^2^College of Sports Engineering and Information Technology, Wuhan Sports University, Wuhan, China

**Keywords:** electroencephalogram, epilepsy, noise robustness, low-rank learning, pinball loss function

## Abstract

Electroencephalogram (EEG) is often used in clinical epilepsy treatment to monitor electrical signal changes in the brain of patients with epilepsy. With the development of signal processing and artificial intelligence technology, artificial intelligence classification method plays an important role in the automatic recognition of epilepsy EEG signals. However, traditional classifiers are easily affected by impurities and noise in epileptic EEG signals. To solve this problem, this paper develops a noise robustness low-rank learning (NRLRL) algorithm for EEG signal classification. NRLRL establishes a low-rank subspace to connect the original data space and label space. Making full use of supervision information, it considers the local information preservation of samples to ensure the low-rank representation of within-class compactness and between-classes dispersion. The asymmetric least squares support vector machine (aLS-SVM) is embedded into the objective function of NRLRL. The aLS-SVM finds the maximum quantile distance between the two classes of samples based on the pinball loss function, which further improves the noise robustness of the model. Several classification experiments with different noise intensity are designed on the Bonn data set, and the experiment results verify the effectiveness of the NRLRL algorithm.

## Introduction

Brain computer interface (BCI) is a system that collects the signals from the brain to communicate with computers or other devices (Gummadavelli et al., 2018; Jiang et al., 2020). As an efficient way for the human brain to directly communicate with peripheral devices, the BCI does not need to rely on the peripheral nervous system and muscles. Electroencephalogram (EEG) signals, as a biomarker, play an important role in BCI. EEG is often used in clinical diagnosis to determine the presence and type of epilepsy (Fahimi et al., 2019; Jiang et al., 2019). The epileptic seizure process has several different periods: interictal, pre-seizure, and seizure. The waveform, frequency, and signal characteristics of different stages are different in EEG. Based on the analysis of the characteristics of epilepsy EEG, many studies can generally be divided into two directions: epilepsy detection and epilepsy prediction (Jiang et al., 2017; Gu et al., 2021). The epilepsy detection algorithm uses signal processing, machine learning, and deep learning to extract signal features, and distinguishes the EEG signals between the interictal period and the seizure period. The epilepsy prediction algorithm distinguishes the EEG signals in the pre-seizure period and the seizure period. The prediction task is more difficult than the detection task. First of all, there is no uniform definition of epilepsy prediction and internal standards in the industry. Secondly, compared with the EEG signal in the seizure period, the signal pattern of the EEG signals in the pre-seizure period and the EEG signals in the intermittent period are more similar, so the algorithm is required to be more robust.

Both epilepsy detection and epilepsy prediction are essentially classification tasks in machine learning (Ni et al., 2020). Several studies focus on the classification of EEG information, which involve both epilepsy detection tasks and epilepsy prediction tasks. Zhou et al. (2018) represented the epilepsy EEG signal into a two-dimensional image, and then they constructed a convolutional neural network to automatically learn the transformed image. This method borrows the idea of image processing to analyze the EEG signals and broaden the method of signal processing. Birjandtalab et al. (2017) performed non-linear dimensionality reduction on EEG signals after extracting time-frequency features. This feature processing method can reflect the non-linear relationship of the data in the process of low-dimensional mapping. Ramakrishnan and Muthanantha (2018) computed the approximate entropy value, the maximum Lyapunov component, and the correlation coefficient dimension on different sub-bands of epilepsy EEG signals, and introduced fuzzy rules to fuzzify the features. The authors believe that fuzzy rules are the natural choice of using human professional knowledge to build machine learning systems, which is closely related to people’s way of thinking. Wang et al. (2017) explored multiple bands of the EEG signal by considering the maximum and standard deviation characteristics of each band. Then they constructed the feature vector of the EEG and used a one-to-one self-organization strategy to create a high high-precision epilepsy detection system. Sun et al. (2019) intercepted and analyzed the pre-seizure data, and they used recurrent autoencoders on multivariate signals to extracted EEG features. Liu et al. (2019) transformed the EEG signal into spectral data through the combination of dimensionality reduction and short-time Fourier transform. Then, the authors constructed a shallow convolutional neural network (CNN) network to automatically learn data features. Yu et al. (2020) used the local mean decomposition (LMD) method to obtain the feature matrix of the EEG signals, and they used a CNN model to implement feature extraction and combined Bayesian linear discriminant analysis to obtain the prediction result.

In supervised learning, the support vector machine (SVM) represented by least squares regression (LSR) is a simple and effective method. The core idea of LSR is to learn the non-linear projection from the original data to the feature space, and the obtained projection vector of the original data is also used as the data representation in the label space. For example, discriminative LSR method include multiclass classification (Xiang et al., 2012), groupwise retargeted LSR method (Ling and Geng, 2019), regularized label relaxation linear regression (Fang et al., 2018), double relaxed regression for classification (Han et al., 2019), and so on. For epilepsy data, the scalp EEG data will have more impurity signals and noise signals. Moreover, dimensional explosion and information redundancy problems are common in EEG signals. Learning a discriminatively compact data representation is a very critical problem in pattern recognition. At present, there are many methods based on subspace learning and least squares classifier to learn good classifiers. For example, to combine projection learning with the task of exploring label information, Meng et al. (2020) proposed a constrained discriminative projection learning for joint optimization of subspace learning and classification problems, which used low-rank constraints to learn robust subspaces to connect the original visual features and target output.

Subspace learning essentially tries to find a suitable low-dimensional space in which the discriminative representation of the original features is preserved as much as possible. In recent years, low-rank learning has achieved relatively good results in matrix analysis, data recovery, and data denoising. At the same time, low-rank representation is an effective means to describe the structure of high-dimensional data, and it is a generalized form of sparsity in matrix space. That is, low-rank representation can describe the low-dimensional subspace structure of high-dimensional data, thus its component in the subspace becomes the most important factor in characterizing the data. In addition, low-rank representation effectively introduces low-rank constraints into the data matrix, which can help to construct discriminative feature subspaces and eliminate outliers. Inspired by this idea, the noise robustness low-rank learning (NRLRL) algorithm is proposed for EEG signal classification. NRLRL learns a low-rank subspace that connects the original data space and the label space. It fully considers the correlation information and local structure of samples, and it guarantees the minimum rank of the coefficient matrix constructed of data under its self-expression. By integrating the multi-class asymmetric least squares SVM classifier with low-rank representation, NRLRL is insensitive to noise and outliers. The experiments performed on noisy EEG signals are shown that our algorithm is noise robust. NRLRL has several advantages as follows: (1) since the low-rank representation follows the minimum rank criterion, NRLRL is robust when reconstructing the original data with noise and outliers. (2) By full use of supervised information and pinball loss function, an asymmetric least square SVM is jointly learned into our objection function, so that NRLRL explores a robust classifier in the framework of low-rank learning. (3) Local constraints based on low-rank representations are used based on supervision information. The criteria of low-rank representations for minimum within-class and maximum between-classes are adopted to capture the discriminative structure of the data.

## Background

### Low-Rank Representation

Give a set of data samples **X** = [**x**_1_,…,**x**_*n*_], each sample **x**_*i*_ ∈ **R***^d^*in **X** can be represented as a linear combination of atoms from a dictionary **A**:

(1) X⁢=⁢AC,

where **C** = [**c**_1_,…,**c**_*n*_] ∈ **R**^*n*×*m*^is the coefficient matrix rank representation.

As a common practice in low-rank learning, the dictionary **A** is set to **X**, i.e.,

(2) X⁢=⁢XC,

Eq. (2) uses the data set itself to represent the data, which is called the self-expression of the data. Each data sample in data set **X** can be represented by:

(3) $${\text{x}}_{i}=\sum_{j\neq i}C_{i\,j}\,{\text{x}}_{j},$$

By minimizing the rank of the coefficient matrix **C**, Eq. (1) can be written by:

(4) $$\begin{array}{c} \min_{C}r\,a\,n\,k\,\left(C\right),\\ s.t.\text{X}\,=\,\text{XC}. \end{array}$$

where*rank*(**C**) is the rank function of **C**.

Considering the existence of noise and outliers in the data sample, the structure of the original data **X** is taken as two parts: one is the linear combination of the dictionary **X** and a low-rank coefficient matrix **C**, and the second part is noise (error) matrix, i.e.,

(5) X⁢=⁢XC⁢+⁢E,

Then the low-rating representation can be defined as follows:

(6) $$\begin{array}{c} \min_{C,E}r\,a\,n\,k\,\left(C\right)+\mu \,{||E||}_{2,0},\\ s.t.\text{X}=\text{XC}\,+\,\text{E}, \end{array}$$

where ||⋅||_2,0_means the ℓ_*2,0*_-norm operator. μ is the trade-off parameter. In Eq. (6), the term ||*E*||_2,0_ encourages the sparseness of the error components.

The low-rank optimization problem of Eq. (6) is a non-convex NP-hard problem. To find its unique optimal solution, it is necessary to perform convex relaxation of Eq. (6). The kernel norm is the best convex approximation of the rank function on the unit sphere in the matrix spectral norm (Candès and Recht, 2009). Therefore, the convex kernel norm can be used to approximate the non-convex rank function, and the ℓ_*2,0*_ norm can be relaxed to its ℓ_*2,1*_norm (Raghunandan et al., 2010). Then Eq. (6) can be written as the following convex optimization problem

(7) $$\begin{array}{c} \min_{C,E}{||C||}_{*}+\mu \,{||E||}_{2,1},\\ s.t.\text{X}=\text{XC}+\text{E}, \end{array}$$

where ||⋅||_*_ means the nuclear norm operator, and||⋅||_2,1_means the ℓ_*2,1*_-norm operator.

### Asymmetric Least Squares Support Vector Machine

The loss function in the least squares SVM (LS-SVM) pays attention to both the correctly classified and incorrectly classified samples. It minimizes the squared error of the classifier as follows,

(8) $$l\,\left(x\right)={\left(1-{\text{w}}^{T}\,\text{x}\text{ –b}\right)}^{2}.$$

In fact, the above loss function is noise sensitive, especially the noises around the separation hyperplane. Many extensions of least squares loss function have been proposed to solve this problem, such as iteratively reweighted least square (Leski, 2015) and asymmetric square function (Leski, 2015), asymmetric squared loss (Huang et al., 2014). Using the statistical property to lower quantile value, the asymmetric squared loss is defined as:

(9) Lpa⁢L⁢S⁢(x)={p⁢(1-wT⁢x+b)2,i⁢f⁢ 1-wT⁢x+b≥0(1-p)⁢(1-wT⁢x+b)2,i⁢f⁢ 1-wT⁢x+b<0

where **w** and *b* are the hyperplane parameter and bias parameter of SVM classifier, respectively. *p* is the lower quantile value parameter.

The aLS-SVM uses the expectile distance and maximizes the expectile distance between different classes. The aLS-SVM has the following optimization problem:

(10) $$\min_{w,b}=\frac{1}{2}\,{\text{w}}^{T}\,\text{w}+\frac{\alpha }{2}\,\sum_{i=1}^{n}L_{p}^{a\,L\,S}\,{\left(1-y_{i}\,\left({\text{w}}^{T}\,{\text{x}}_{i}+b\right)\right)}^{2}.$$

where α is the regularization parameter. This optimization problem can be solved by quadratic programming method.

## Noise Robustness Low-Rank Learning Algorithm

### The Noise Robustness Low-Rank Learning Model

Given a set of data points **X** = [**x**_1_,…,*x*_*n*_] and their labels **Y** = [**y**_1_,…,**y**_*n*_] are distributed in *K* classes. **y**_*k*_ = [*y*_1,*k*_,*y*_2,*k*_,…,*y*_*n*,*k*_] is the class label vector of *n* training samples associated with the *k*-th class. Considering the influence of noise or outliers, the main goal of our algorithm is to find the lowest rank representation **C** and the best classifier based on **C**.

First, to increase discrimination capability, the local preservation with label embedding is incorporated into the learning process. Different from the traditional local preservation term in low-rank learning, the label information is embedded into the *k*-nearest neighborhood relationships. For sample **x**_*i*_, its low-rank is expressed as **c**_*i*_. Without considering the label information of the sample, if **x**_*j*_ is in the *k*-nearest neighbor of **x**_*i*_, their corresponding low-rank representations **c**_*j*_ and **c**_*i*_ should be closer to each other. Obviously, this strategy is not suitable for classification tasks. Based on the basic classification principles of within-class compactness and between-classes separation, the label information is introduced into the *k*-nearest neighborhood relationships. The within-class matrix **B**_*within*_ and between-classes matrix **B**_*between*_ are accordingly defined, and their elements can be defined as,

(11) $$B_{w\,i\,t\,h\,i\,n,i\,j}=\left\{ \begin{array}{c} \exp\left(-{||{\text{x}}_{i}-{\text{x}}_{j}||}^{2}/t\right),i\,f\,{\text{x}}_{i}\in N\,\left({\text{x}}_{j}\right)\,o\,r\,{\text{x}}_{j}\in N\,\left({\text{x}}_{i}\right),\\ y_{i}=y_{j},\\ 0,o\,t\,h\,e\,r\,w\,i\,s\,e\; \end{array}\right.$$

(12) $$B_{b\,e\,t\,w\,e\,e\,n,i\,j}=\left\{ \begin{array}{c} -\exp\left(-{||{\text{x}}_{i}-{\text{x}}_{j}||}^{2}/t\right),i\,f\,{\text{x}}_{i}\in N\,\left({\text{x}}_{j}\right)\,o\,r\\ x_{j}\in N\,\left({\text{x}}_{i}\right),y_{i}\neq y_{j},\\ 0,o\,t\,h\,e\,r\,w\,i\,s\,e\; \end{array}\right.$$

where *N*(**x**_*j*_) returns the *k*-nearest neighbors of **x**_*j*_.

In the NRLRL, the original data is projected into a low-dimensional subspace by low-rank representation. NRLRL shows the similarity within the class and the difference between classes of the data. To achieve this goal, the label embedded local preservation term is defined as,

(13) $$\begin{array}{c} \min_{C}=\frac{1}{2}\,\sum_{i=1}^{n}\sum_{j=1}^{n}{||{\text{c}}_{i}-{\text{c}}_{j}||}_{2}^{2}\,\left(B_{w\,i\,t\,h\,i\,n,i\,j}-B_{b\,e\,t\,w\,e\,e\,n,i\,j}\right)\\ =T\,r\,\left({\text{CB}}_{w\,i\,t\,h\,i\,n}\,{\text{C}}^{T}\right)-T\,r\,\left({\text{CB}}_{b\,e\,t\,w\,e\,e\,n}\,{\text{C}}^{T}\right)\\ =T\,r\,\left({\text{CLC}}^{T}\right) \end{array}$$

where **L** = **L***_between_* − **L***_within_*, **L***_between_* = **B***_between_* − **B***_between_*, **L***_within_* = **B***_within_* − **B***_within_*. **B***_between_* and **B***_within_* are diagonal matrices, and their elements are ${\text{B}}_{b\,e\,t\,w\,e\,e\,n,i\,i}=\sum_{j=1}^{n}{\text{B}}_{b\,e\,t\,w\,e\,e\,n,i\,j}$, ${\text{B}}_{w\,i\,t\,h\,i\,n,i\,i}=\sum_{j=1}^{n}{\text{B}}_{w\,i\,t\,h\,i\,n,i\,j}$, separately. *Tr*(⋅) is the trace operator.

The label embedded local preservation term is to ensure that if the nearest neighbors **x**_*i*_ and **x**_*j*_ are from the same class, their low-rank codes **c**_*i*_ and **c**_*j*_ are also close to each other. At the same time, if the nearest neighbors **x**_*i*_ and **x**_*j*_ are from different classes, their low-rank codes **c**_*i*_ and **c**_*j*_ are separated as much as possible. In such a low-rank learning stage, the ideal non-linear local structure of EEG data is preserved.

To promote the discriminative ability of low-rank representation vectors, a multi-class aLS-SVM classification term is embedded into the NRLRL algorithm. The multi-class aLS-SVM classification term **L**(*C*)includes two parts

(14) $$\begin{array}{c} L\,\left(C,W,b\right)=\sum_{k=1}^{K}\left(f\,\left(C,{\text{y}}_{k},{\text{w}}_{k},b_{k}\right)+\alpha \,{||{\text{w}}_{k}||}_{2}^{2}\right),\\ f\,\left(C,{\text{y}}_{k},{\text{w}}_{k},b_{k}\right)=\sum_{i=1}^{n}l\,\left({\text{c}}_{i},y_{k,i},{\text{w}}_{k},b_{k}\right), \end{array}$$

where *l*(**c**_*i*_,*y*_*k*,*i*_,**w**_*k*_,*b*_*k*_) is the loss function associated with the *k*-th aLS-SVM.

The squared pinball loss in NRLRL can be written as

(15) $$l\,\left(\mu \right)=\left\{ \begin{array}{c} p\,\mu^{2},\mu \geq 0,\\ \left(1-p\right)\,\mu^{2},\mu <0, \end{array}\right.$$

where $\mu_{k,i}=y_{k,i}\,\left({\text{w}}_{k}^{T}\,{\text{c}}_{i}+b_{k}\right)-1$.

Bedding the local preservation with label embedding term and multi-class aLS-SVM classification term into the Eq.(7), the objective function of NRLRL can be written as

(16) $$\begin{array}{c} \min_{C,E,W,b}{||C||}_{*}+\lambda \,{||E||}_{2,1}+\gamma \,L\,\left(C,W,b\right)+\eta \,T\,r\,\left({\text{CLC}}^{T}\right),\\ s.t.\text{X}=\text{XC}+\text{E},\\ 1_{n}^{T}\,\text{C}=1_{n}^{T}, \end{array}$$

where λ,γ, and η are regularization parameters.

The loss function term is decomposed into the sum of the loss term of each sample, and it can be seen that the contribution of each data sample to the objective function is linearly cumulative. *L*(**C**,**W**,**b**) includes a class-by-class loss function term *l*(**c**_*i*_,*y*_*k*,*i*_,**w**_*k*_,*b*_*k*_)on the low-rank representation, so that the obtained lowest-rank representations are highly correlated within the class. EEG signals belonging to the same class usually contain the common discriminative features. The lowest-rank representation obtained by Eq. (16) has the characteristics of strong within-class correlation and between-class difference for classification tasks.

To reduce the time costs, the Frobenius norm is used to replace the nuclear norm, the objective function of NRLRL can be re-written as:

(17) $$\begin{array}{c} \min_{C,E,W,b}{||C||}_{F}^{2}+\lambda \,{||E||}_{2,1}+\gamma \,L\,\left(C,W,b\right)+\eta \,T\,r\,\left({\text{CLC}}^{T}\right),\\ s.t.\text{X}=\text{XC}+\text{E},\\ 1_{n}^{T}\,\text{C}=1_{n}^{T}, \end{array}$$

For simplicity of expression, combining the two terms ${||C||}_{F}^{2}$ and *Tr*(*C^T^LC*), Eq. (17) can be written as:

(18) $$\begin{array}{c} \min_{C,E,W,b}\lambda \,{||E||}_{2,1}+\gamma \,L\,\left(C,W,b\right)+T\,r\,\left(\text{C}\,\left(\eta \,\text{L}+\text{I}\right)\,{\text{C}}^{T}\right),\\ s.t.\text{X}=\text{XC}+\text{E},\\ 1_{n}^{T}\,\text{C}=1_{n}^{T}, \end{array}$$

From Eq. (18), we can see that the NRLRL algorithm consists of three sub-problems, namely the parameters of **C**, **E**, and aLS-SVM classifier. These three sub-problems can be solved alternately until the NRLRL algorithm converges. We use the alternating direction multipliers method (ADMM) (Luo et al., 2017) to solve Eq. (18). The augmented Lagrangian function corresponding to Eq. (18) can be written as:

(19) $$\begin{array}{c} \min_{C,E,W,b}\lambda \,{||E||}_{2,1}+\gamma \,L\,\left(C,W,b\right)+T\,r\,\left(\text{C}\,\left(\eta \,\text{L}+\text{I}\right)\,{\text{C}}^{T}\right)\\ +T\,r\,\left[\theta^{T}\,\left(\text{X}-\text{XC}-\text{E}\right)\right]+T\,r\,\left[\delta^{T}\,\left(1_{n}^{T}\,\text{C}-1_{n}^{T}\right)\right]\\ +\frac{\mu }{2}\,\left({||\text{X}\text{ -}\text{XC}-\text{E}||}_{F}^{2}+{||1_{n}^{T}\,\text{C}-1_{n}^{T}||}_{F}^{2}\right) \end{array}$$

where θ and δ are Lagrange multipliers, and μ is the penalty parameter.

### Optimization of the Objective Function

According to the ADMM algorithm, the parameters in Eq. (19) can be updated alternately, that is, when one parameter is updated, other parameters are fixed until the NRLRL algorithm converges.

(1) Update **C** by fixing **E**, **w***_*k*_*, and *b*_*k*_. Eq. (19) is converted to the following problem:

(20) $$\begin{array}{c} L\,\left({\text{c}}_{i}\right)=\gamma \,{||1-y_{k,i}\,\left({\text{w}}_{k}^{T}\,{\text{c}}_{i}+b_{k}\right)||}_{2}^{2}+T\,r\,\left({\text{c}}_{i}\,\left(\eta \,\text{L}+\text{I}\right)\,{\text{c}}_{i}^{T}\right)\\ +T\,r\,\left[\theta^{T}\,\left({\text{x}}_{i}-{\text{x}}_{i}\,{\text{c}}_{i}\right)+\delta^{T}\,1_{n}^{T}\,{\text{c}}_{i}\right]\\ +\frac{\mu }{2}\,\left({||{\text{x}}_{i}-{\text{x}}_{i}\,{\text{c}}_{i}-{\text{e}}_{i}||}_{F}^{2}+{||1_{n}^{T}\,{\text{c}}_{i}-1_{n}^{T}||}_{F}^{2}\right). \end{array}$$

Let the derivative of Eq. (19) with respect to *c*_*i*_ be zero, the solution of *c*_*i*_ is:

(21) $${\text{c}}_{i}={\left(2\,\left(\eta \,\text{L}+\text{I}\right)+\mu \,\left({\text{x}}_{i}^{T}\,{\text{x}}_{i}+1_{n}\,1_{n}^{T}\right)+2\,\gamma \,\sum {\text{w}}_{k}\,{\text{w}}_{k}^{T}\right)}^{-1}(\mu \left({\text{x}}_{i}^{T}{\text{x}}_{i}-{\text{x}}_{i}^{T}{\text{e}}_{i}+1_{n}1_{n}^{T}\right)+{\text{x}}_{i}^{T}\theta -1_{n}\delta +2\gamma \sum {\text{w}}_{k}\left(y_{k,i}-b_{k}\right))$$

(2) Update **E** by fixing **C**, **w***_*k*_*, and *b*_*k*_. Using the same calculation and reduction strategy by Liu et al. (2013). Eq. (19) is converted to the following problem:

(22) $$\min_{\text{E}}\frac{\lambda }{\mu }\,{||E||}_{2,1}+\frac{1}{2}\,{||\text{E}-\left(\text{X}-\text{XC}+\frac{1}{\mu }\,\theta \right)||}_{F}^{2},$$

Then the solution of **E** can be obtained by:

(23) $$\text{E}\,\left(:,i\right)=\left\{ \begin{array}{c} \frac{||\theta_{i}||-\lambda }{||\theta_{i}||}\,\theta_{i},i\,f\,\frac{\lambda }{\mu }<||\theta_{i}||,\\ 0,o\,t\,h\,e\,r\,w\,i\,s\,e \end{array}\right.$$

where θ_*i*_is the *i*th column vector of the matrix θ.

(3) Update **w***_*k*_* and *b*_*k*_ by fixing **E** and **C**. Eq. (19) is converted to the following problem:

(24) $$\min_{w_{k},b_{k}}\sum_{k=1}^{K}\left\{\alpha |{\left|{\text{w}}_{k}\right||}_{2}^{2}+\sum_{i=1}^{n}l\,\left({\text{c}}_{i},y_{i,k},{\text{w}}_{k},b_{k}\right)\right\},$$

Eq. (24) is a multi-class aLS-SVM problem, and the optimal parameters **w***_*k*_* and *b*_*k*_ can be obtained by the aLS-SVM algorithm.

The training procedure of NRLRL is summarized in Algorithm 1.

**ALGORITHM 1:** The training procedure of NRLRL is summarized.

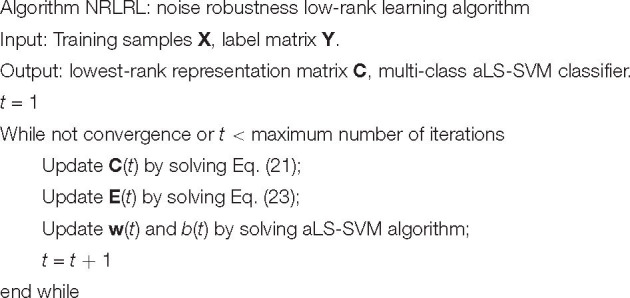

## Experiment

### Datasets and Experimental Settings

**FIGURE 1:**
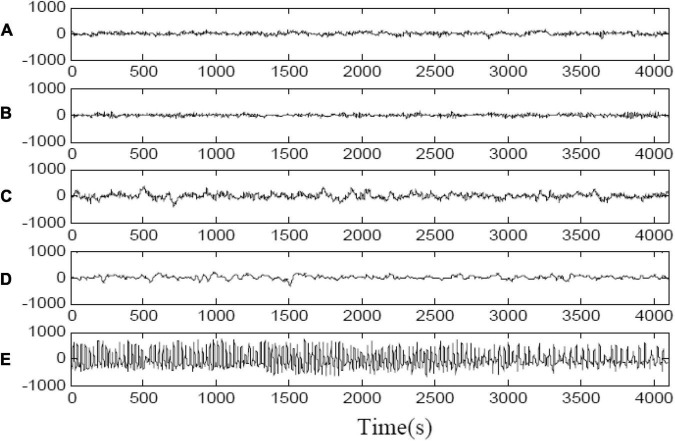
Sample electroencephalogram (EEG) signals in each group in Bonn dataset. **(A)** Epileptic EEG signals measured from healthy people with eyes open. **(B)** Epileptic EEG signals measured from healthy people with eyes closed. **(C)** Epileptic EEG signals obtained in hippocampal formation of the opposite hemisphere of brain during seizure free intervals. **(D)** Epileptic EEG signals obtained from within epileptogenic zone during seizure free intervals. **(E)** Epileptic EEG signals measured during seizure activity.

This study used EEG signals are from Bonn University. The data set consists of five subsets (groups A to E), each of which consists of 100 EEG segments with a single channel duration of 23.6 s and 4,097 samples. The fragments in groups A-B were taken from five healthy subjects, and the fragments in groups {C, D, E} were taken from patients with epilepsy. The groups C and D recorded the signal during the intermittent period of epileptic seizures. The group E recorded the signal during the seizure. The signals of the five groups of EEG data are shown in Figure 1. In the experiment, the 4,097 data points were divided into three data blocks to obtain the research samples, that is, a data block is a sample, representing the EEG information in about 8 s. Therefore, the sample size of this paper is 3 × 100 = 300 in each group, and each sample has 1,365 features of sampling points. We design two types of classification tasks on the Bonn dataset. One is the binary classification task: non-epileptic condition (sets {A, B, C, D, E}) and epileptic condition (set E). The other is the three classes of classification task: normal (sets {A, B}), interictal (sets {C, D}), and ictal (set E).

We use the following algorithms as the comparison algorithms: DLSR (Xiang et al., 2012), LC-KSVD (Jiang et al., 2013), SRRS (Li and Fu, 2016), LRSD (Kong et al., 2017), aLS-SVM (Huang et al., 2014), and LRDLSR (Chen et al., 2020). The parameters of these comparison methods are set to their default settings. In NRLRL, the dictionary size is set from {40, …, 320}, three regularization parameters are set from {2^–5^, …, 2^3^}, the *k*-nearest neighbor parameter is set from {3, …, 11}, and the pinball loss parameter *p* is set from {40, …, 360}. We adopt the one-by-one strategy to select the optimal parameters.

Following the method of references (Huang et al., 2014; Gu et al., 2019, 2020), 20 and 50% samples are randomly selected and common Gaussian white noise is added. To test the sensitivity of the classifier to noise intensity, the intensity of Gaussian white noise is divided into three types: the mean value is 0, and the variance is set to 5, 10, and 15% of the sample features, respectively. For example, the *noise* (20%, 10%) indicates that 20% of the samples in the Bonn dataset contain Gaussian white noise, and the variance of the noise is 10% of the sample features.

### Classification Result Comparison

First, we perform experiments on the binary classification task. We compare all algorithms in indexes of specificity, sensitivity, and accuracy on the noisy Bonn dataset. The experimental results of the binary classification task are shown in Tables 1–3.

**TABLE 1:** The specificity results of binary classification task on the noisy Bonn dataset.

	DLSR	LC-KSVD	SRRS	LRSD	aLS-SVM	LRDLSR	NRLRL
*noise* (20%, 5%)	92.93	93.06	95.08	95.06	95.57	95.50	**96.55**
*noise* (20%, 10%)	92.61	92.70	95.05	95.21	95.32	95.61	**96.53**
*noise* (20%, 15%)	92.04	92.87	95.06	95.01	94.80	95.46	**96.51**
*noise* (50%, 5%)	90.97	91.11	94.61	94.96	95.06	95.30	**96.40**
*noise* (50%, 10%)	90.40	90.54	94.36	94.29	94.62	95.23	**96.36**
*noise* (50%, 15%)	89.84	90.32	93.99	94.31	94.49	95.06	**96.33**

*The bold values mean the best values in comparison experiments.*

**TABLE 2:** The sensitivity results of binary classification task on the noisy Bonn dataset.

	DLSR	LC-KSVD	SRRS	LRSD	aLS-SVM	LRDLSR	NRLRL
*noise* (20%, 5%)	92.94	93.49	95.31	95.50	95.74	95.96	**96.65**
*noise* (20%, 10%)	92.63	93.18	95.24	95.39	95.52	95.91	**96.63**
*noise* (20%, 15%)	92.08	92.91	95.27	95.25	95.40	95.84	**96.58**
*noise* (50%, 5%)	90.04	91.63	94.71	95.11	95.28	95.54	**96.52**
*noise* (50%, 10%)	90.42	91.02	94.52	94.31	94.72	95.31	**96.48**
*noise* (50%, 15%)	89.95	90.47	94.16	94.39	94.77	95.15	**96.46**

*The bold values mean the best values in comparison experiments.*

**TABLE 3:** The accuracy results of binary classification task on the noisy Bonn dataset.

	DLSR	LC-KSVD	SRRS	LRSD	aLS-SVM	LRDLSR	NRLRL
*noise* (20%, 5%)	92.93	93.22	95.24	95.20	95.57	95.69	**96.58**
*noise* (20%, 10%)	92.61	92.72	95.16	95.33	95.34	95.76	**96.57**
*noise* (20%, 15%)	92.07	92.88	95.19	95.19	94.99	95.48	**96.54**
*noise* (50%, 5%)	90.03	91.29	94.62	95.02	95.18	95.33	**96.42**
*noise* (50%, 10%)	90.41	90.69	94.47	94.28	94.63	95.26	**96.41**
*noise* (50%, 15%)	89.90	90.46	94.06	94.38	94.57	95.09	**96.39**

*The bold values mean the best values in comparison experiments.*

From the experimental results, it can be seen that (1) with the increase of noise intensity, the specificity, sensitivity, and accuracy of all algorithms show a decline in varying degrees. It can be seen that the characteristic noise of samples will seriously affect the classification effect of the classifier. Especially the DLSR and LC-KSVD algorithms do not consider the impact of the noise sample interference on the classification surface. As the noise intensity increases, the classification result decreases rapidly. (2) SRRS, LRSD, aLS-SVM, LRDLSR, and NRLRL algorithms are all noise-insensitive classification algorithms, therefore the classification results are significantly better than conventional classification algorithms. The proposed NRLRL algorithm achieves the best results in classification performance. The NRLRL algorithm removes the influence of noise on the sample in the lowest rank representation, and it uses the pinball loss function to obtain a noise-insensitive classification classifier by maximizing the distance between the two classes of quantile distances. In addition, the NRLRL algorithm can mine the geometric structure of samples in low-dimensional space by low-rank learning, and fully considers the correlation information and subspace structure between samples. Therefore, the within-class similarity and between-class differences of the data are more prominent, which makes the NRLRL algorithm obtain good classification performance in the presence of noise.

Then, we perform experiments on three class classification task, i.e., classification of EEG data in normal, interictal and epileptic periods. Similarly to the above experiment procedure, we compare all algorithms in indexes of specificity, sensitivity, and accuracy on the noisy Bonn dataset. The experimental results of three classification task are shown in Tables 4–6. From these results, it can be observed that since the complexity of the three class classification is higher than that of the binary class classification, the results of three class classification are lower than those of binary class classification. The proposed NRLRL algorithm achieves the best results of specificity, sensitivity, and accuracy.

**TABLE 4:** The specificity results of three class classification task on the noisy Bonn dataset.

	DLSR	LC-KSVD	SRRS	LRSD	aLS-SVM	LRDLSR	NRLRL
*noise* (20%, 5%)	89.41	89.43	92.45	92.78	92.66	93.31	**94.18**
*noise* (20%, 10%)	89.20	89.15	91.06	92.26	91.65	93.27	**94.11**
*noise* (20%, 15%)	88.34	88.45	91.72	92.04	91.90	93.08	**93.96**
*noise* (50%, 5%)	87.59	87.28	92.92	91.77	91.27	92.41	**93.69**
*noise* (50%, 10%)	86.85	86.87	91.67	91.46	92.19	92.44	**93.62**
*noise* (50%, 15%)	85.30	85.35	91.41	91.13	91.15	92.83	**93.50**

*The bold values mean the best values in comparison experiments.*

**TABLE 5:** The sensitivity results of three class classification task on the noisy Bonn dataset.

	DLSR	LC-KSVD	SRRS	LRSD	aLS-SVM	LRDLSR	NRLRL
*noise* (20%, 5%)	89.51	89.51	92.60	92.85	92.69	93.39	**94.20**
*noise* (20%, 10%)	89.22	89.26	92.1	92.31	92.74	93.32	**94.14**
*noise* (20%, 15%)	88.66	88.49	91.99	92.03	92.08	93.27	**93.99**
*noise* (50%, 5%)	87.69	87.33	92.53	91.78	91.42	92.55	**93.72**
*noise* (50%, 10%)	86.88	86.81	91.79	91.51	92.21	92.54	**93.65**
*noise* (50%, 15%)	86.49	86.42	91.49	91.19	91.28	92.85	**93.53**

*The bold values mean the best values in comparison experiments.*

**TABLE 6:** The accuracy results of three class classification task on the noisy Bonn dataset.

	DLSR	LC-KSVD	SRRS	LRSD	aLS-SVM	LRDLSR	NRLRL
*noise* (20%, 5%)	89.46	89.48	92.48	92.80	92.67	93.32	**94.18**
*noise* (20%, 10%)	89.20	89.17	92.04	92.28	92.61	93.29	**94.01**
*noise* (20%, 15%)	88.54	88.48	91.77	92.03	91.92	93.19	**93.95**
*noise* (50%, 5%)	87.57	87.28	92.47	91.64	91.37	92.49	**93.70**
*noise* (50%, 10%)	86.86	86.82	91.63	91.43	92.18	92.47	**93.63**
*noise* (50%, 15%)	86.40	86.39	91.43	91.14	91.22	92.82	**93.52**

*The bold values mean the best values in comparison experiments.*

### Parameter Analysis

Here we discuss the key parameters in the NRLRL algorithm on binary classification task *noise* (20%, 10%) and three class classification *noise* (50%, 10%).

**FIGURE 2:**
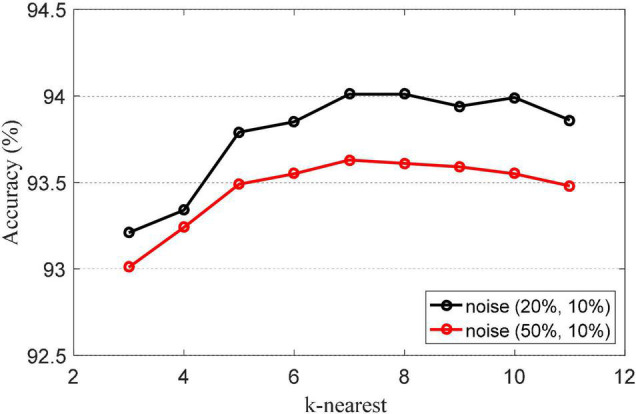
Classification accuracies of noise robustness low-rank learning (NRLRL) with different *k*-nearest.

The *k*-nearest parameter *k*-nearest is an important parameter in NRLRL. It determines the neighbor relationship between samples. The *k*-nearest parameter is set from {3, …, 11}. The classification accuracies of NRLRL with different *k*-nearest are shown in Figure 2. When *k* = 7, the classification accuracy is the highest. The appropriate value of *k* can reflect the local structural information of the sample to the greatest extent. From the results in Figure 2, we can set *k* = 7 in the NRLRL algorithm for noisy Bonn dataset.

**FIGURE 3:**
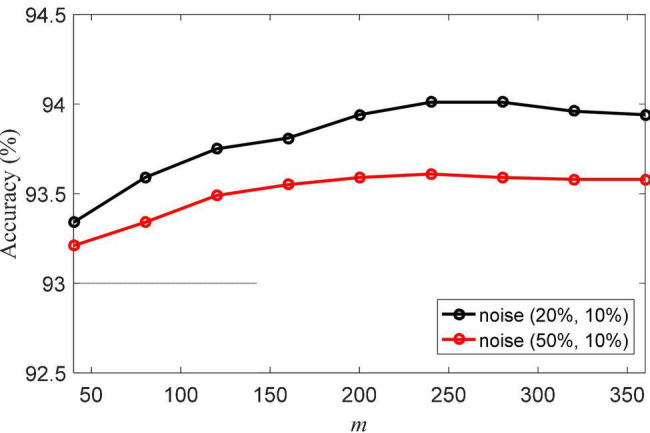
Classification accuracies of noise robustness low-rank learning (NRLRL) with different *m*.

Another important parameter is *m*, which is the size of the matrix **C**. The classification accuracies of NRLRL with different *m* are shown in Figure 3. The parameter *m* controls the data structure of the low-rank space. When *m* is too small, the low-rank representation related to the data is not enough to model its structure in the low-rank space. When *m* is too large, the redundant information will produce errors of low-rank representation. From the results in Figure 3, we can set *m* = 240.

**FIGURE 4:**
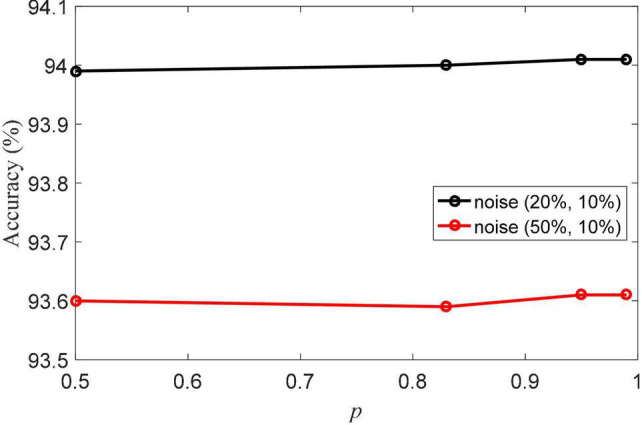
Classification accuracies of noise robustness low-rank learning (NRLRL) with different *p*.

The pinball loss parameter *p* is the important parameter in aLS-SVM classifier in NRLRL. The value range of the pinball loss parameter is {0.5, 0.83, 0.95, 0.99}. The classification accuracies of NRLRL with different *p* are shown in Figure 4. With different values of *p*, the NRLRL algorithm has achieved high classification accuracy. It shows that the NRLRL algorithm is not sensitive to the *p* parameter, so the value of *p* can be fixed to 0.95 in the experiment.

## Conclusion

In this study, the NRLRL algorithm is proposed for EEG signal classification. Different from noise-insensitive SVM learns the classification hyperplane in the original space or kernel space, NRLRL learns a low-rank subspace as the transformation from the original data space to the label space, to improve the overall classification effect. By introducing the criteria of low-rank representations for minimum within-class and maximum between-classes, the discriminative ability of the model has been greatly improved. The pinball loss function is also helpful to improve the noise insensitivity of the model. The effectiveness of the proposed algorithm is verified on the noisy Bonn EEG dataset. Since our algorithm directly uses the EEG sample point as the input features, we will consider applying various feature extraction methods to the NRLRL algorithm in the next stage. The seizure data is often insufficient in epilepsy detection or prediction tasks. To obtain an effective algorithm model, the down-sampling strategy is often performed to make the class balanced. This strategy will cause the loss of signal data. Therefore, research on appropriate imbalanced data classification methods is the focus of the next stage.

## Data Availability Statement

Publicly available datasets were analyzed in this study. This data can be found here: https://github.com/benfulcher/hctsaTutorial_BonnEEG.

## Author Contributions

MG conceived and designed the proposed model and wrote the manuscript. RL and JM performed the experiment. All authors read and approved the manuscript.

## Conflict of Interest

The authors declare that the research was conducted in the absence of any commercial or financial relationships that could be construed as a potential conflict of interest.

## Publisher’s Note

All claims expressed in this article are solely those of the authors and do not necessarily represent those of their affiliated organizations, or those of the publisher, the editors and the reviewers. Any product that may be evaluated in this article, or claim that may be made by its manufacturer, is not guaranteed or endorsed by the publisher.
